# Computational Approaches to Evaluate the Acetylcholinesterase Binding Interaction with Taxifolin for the Management of Alzheimer’s Disease

**DOI:** 10.3390/molecules29030674

**Published:** 2024-01-31

**Authors:** Varish Ahmad, Ibrahim Alotibi, Anwar A. Alghamdi, Aftab Ahmad, Qazi Mohammad Sajid Jamal, Supriya Srivastava

**Affiliations:** 1 Health Information Technology Department, The Applied College, King Abdulaziz University, Jeddah 21589, Saudi Arabiaabdulsalam@kau.edu.sa (A.A.); 2 Pharmacovigilance and Medication Safety Unit, Centre of Research Excellence for Drug Research and Pharmaceutical Industries, King Abdulaziz University, Jeddah 21589, Saudi Arabia; 3 Department of Health Informatics, College of Public Health and Health Informatics, Qassim University, Al Bukayriyah 52741, Saudi Arabia

**Keywords:** taxifolin, cholinesterase inhibitor, Alzheimer’s disease, neurodegenerative disorders, molecular dynamics simulation

## Abstract

Acetylcholinesterase (AChE) and butyrylcholinesterase (BChE) are enzymes that break down and reduce the level of the neurotransmitter acetylcholine (ACh). This can cause a variety of cognitive and neurological problems, including Alzheimer’s disease. Taxifolin is a natural phytochemical generally found in yew tree bark and has significant pharmacological properties, such as being anti-cancer, anti-inflammatory, and antioxidant. The binding affinity and inhibitory potency of taxifolin to these enzymes were evaluated through molecular docking and molecular dynamics simulations followed by the MMPBSA approach, and the results were significant. Taxifolin’s affinity for binding to the AChE–taxifolin complex was −8.85 kcal/mol, with an inhibition constant of 326.70 nM. It was observed to interact through hydrogen bonds. In contrast, the BChE–taxifolin complex binding energy was observed to be −7.42 kcal/mol, and it was significantly nearly equal to the standard inhibitor donepezil. The molecular dynamics and simulation signified the observed interactions of taxifolin with the studied enzymes. The MMPBSA total free energy of binding for AChE–taxifolin was −24.34 kcal/mol, while BChE–taxifolin was −16.14 kcal/mol. The present research suggests that taxifolin has a strong ability to bind and inhibit AChE and BChE and could be used to manage neuron-associated problems; however, further research is required to explore taxifolin’s neurological therapeutic potential using animal models of Alzheimer’s disease.

## 1. Introduction

Alzheimer’s disease (AD) is a neurodegenerative disease that causes symptoms of severe dementia, neurodegeneration, and even Parkinson’s, severely compromising a person’s ability to perform daily activities. AD affects 50–60% of people with dementia; furthermore, this number will increase from 55 million to 151 million [1]. Clinical evidence indicates that AD is a complex disease characterized by inflammation, oxidative stress, abnormal protein levels, memory impairment, behavioral disorders, cognitive impairment, and eventual death [2]. Although the etiology of AD is not fully understood, many factors such as acetylcholine (ACh) deficiency, β-amyloid (β) accumulation, oxidative stress, dysregulation of bio metal homeostasis, and neuro-inflammation have been implicated in AD, suggesting they are important in disease progression.

The cholinergic hypothesis proposes that the degeneration of cholinergic neurons and the effects of cholinergic neurotransmission in the cerebral cortex are responsible for cognitive impairment in the brains of AD patients [3]. Acetylcholinesterase (AChE) and butyrylcholinesterase (BChE) are cholinesterases (ChEs) that hydrolyze ACh in the brain. AChE is mainly derived from synaptic junctions and regions with strong activity in the adult cerebral cortex, whereas BChE is mainly derived from brain glial cells, which are effectively spatially co-stored and promote BChE-mediated hydrolysis, thereby regulating local ACh levels to maintain normal cholinergic function. However, BChE-derived products are shown in pathological conditions [4]. Loss of cholinergic activity can be affected or influenced by many factors such as amyloid peptide production and accumulation, stress, and iron overloading. Surprisingly, AChE inhibitors have been shown to affect the “amyloid cascade”, which begins with accumulating insoluble amyloid-β in the brain. However, AChE can also produce peptide A, which speeds up this process. This activity was reported in the peripheral anion region (PAS) [5]. The activity of these two enzymes must be controlled to manage neuron-associated problems, with natural resources such as plants being excellent sources for discovering and developing new active therapeutic molecules.

Various plants, such as olive, tea, blueberry, strawberry, mint, walnut, helichrysum, and sage, have been shown to demonstrate AChE inhibitory activity due to their polyphenols. In isolation, Turmeric, (-)-epigallocatechin-3-gallate (EGCG), and several flavonoids are also potent AChE inhibitors [6]. Bisphenols have specific patterns of inhibitory activity and can block AChE or BChE. According to Chan et al., caffeic acid and quinic acid do not inhibit AChE or BChE; however, chlorogenic acid and 3-O-caffeoylquinic acid do [7]. The polyphenolic compound (quercetin) that helps prevent Alzheimer’s disease is found in apples. Curcumin, the main active phenolic compound in green tea, along with EGCG and resveratrol, have been associated with AChE inhibition [8]. The flavanone naringenin, an important flavonoid in citrus, has been shown to exhibit AChE inhibitory activity in vitro and amnestic protection in vivo [9]. Although the inhibitory effect of the flavanol quercetin has not been studied in vivo, it also appears to affect cholinergic dysfunction and cerebral blood flow in the brain.

Next-generation AChE inhibitors have attracted interest among researchers, and potential candidates derived from a non-alkaloid class of molecules such as flavonoids have been identified [10]. However, they provide only temporary and incomplete relief of symptoms. In addition, previous studies have shown that AChE can cause amyloid plaques, and the expression of BChE is associated with A β plaques, Neurofibrillary Tangle (NFT), and cerebral amyloid angiopathy.

To date, three of the four drugs have been approved for the treatment of Alzheimer’s disease as an AChE inhibitor. Available drugs, namely, donepezil, galantamine, and rivastigmine, have limitations in efficacy and duration of action. Moreover, AD drugs only reduce the symptoms of dementia and do not stop the development of the degenerative process [11].However, selective BChE inhibitors are mostly so-called irreversible carbamates and tacrine- or donepezil-based hybrids, meaning scientists are limited to discovering new drugs by changing simple structures [12]. Thus, the lack of a current treatment has exacerbated the complications of AD, accelerating the current research searching for new cholinesterase inhibitors from natural resources, including plants. AChE and BChE are still the most important targets for discovering new cholinesterase inhibitors for use as anti-AD drugs. Therefore, new AChE inhibitors need to be developed.

Dihydroquercetinis, a type of flavanonol compound commonly found in citrus fruits, onions, green tea, olive oil, and plants such as *milk thistle*, *French conch*, *Douglas fir* bark, and *Sarsaparilla* [13]. This molecule is also widely used as a food supplement and can be found in medicinal products such as *silymarin*. Taxifolin is gaining increasing attention as a treatment for various ailments, such as cancer, heart disease, viral hepatitis, dyslipidemia, and neurodegenerative diseases [14]. It has many defensive medicinal properties, including anti-oxidation [15], inhibition of advanced glycation end products [16], and mitochondrial protection [17].

Thus, Taxifolin has emerged as an effective and safe therapeutic agent for preventing and treating many diseases, including cancers, but its interaction with ChE is little explored. Moreover, taxifolin offers some advantages over the currently used drugs, including less toxicity. Therefore, a computational investigation was undertaken to identify safe and potent cholinesterase inhibitors and explore taxifolin’s therapeutic potential in the context of neuron disease or disorders. The inhibitory or binding effect of the selected ligand, taxifolin, was evaluated with target enzyme proteins, namely, AChE and BChE.

## 2. Results and Discussion

In this study, the inhibitory potential of taxifolin against cholinesterase enzymes was evaluated through docking, dynamics and simulations in terms of binding energy, inhibition constant, and involved bond. These observed results are discussed in the following sections.

### 2.1. Molecular Docking Analysis

**Figure 1:**
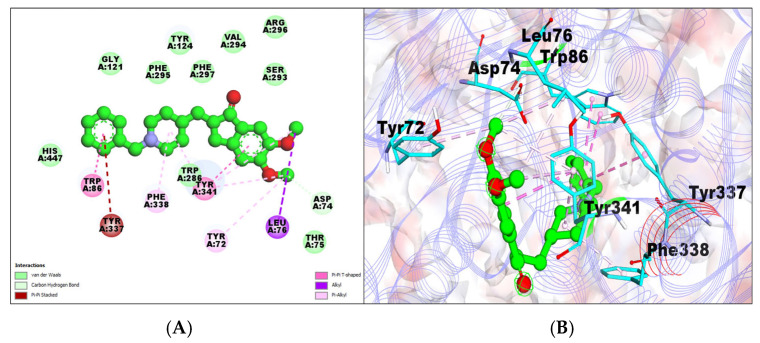
(**A**) A 2D representation of AChE–donepezil (green colored ball and stick pattern in the center) interaction. The interacting amino acid residues are in the surroundings with different color spheres; furthermore, dotted lines show the formation of bonds. (**B**) A 3D representation of observed pose of AChE–donepezil complex. AChE (shown by purple color in line ribbon pattern). Interacting amino acid residues are shown by stick pattern (cyan color) in the surrounding area.

The docking analysis data show that the standard drug donepezil and the tested drug taxifolin significantly interacted with AChE and BChE with different binding affinities. Donepezil has a higher binding affinity for AChE than BChE, while taxifolin has a higher binding affinity for BCHE than AChE (Table 1). The AChE–donepezil complex showed a −9.33 kcal/mol binding affinity with a 144.37 nM inhibition constant and formed one hydrogen bond, i.e., donepezil’s 4-difluorophenyl group forms a hydrogen bond with the Asp residue at position 74 of AChE (Table 1). Amino acid residues TYR72, TYR341, LEU289, GLU292, VAL294, PHE295, PHE338, and PHE297 formed van der Waals interactions. It was also observed that TYR124 and TRP286 are involved in pi–pi stacking (Table 1; Figure 1A,B).

**Figure 2:**
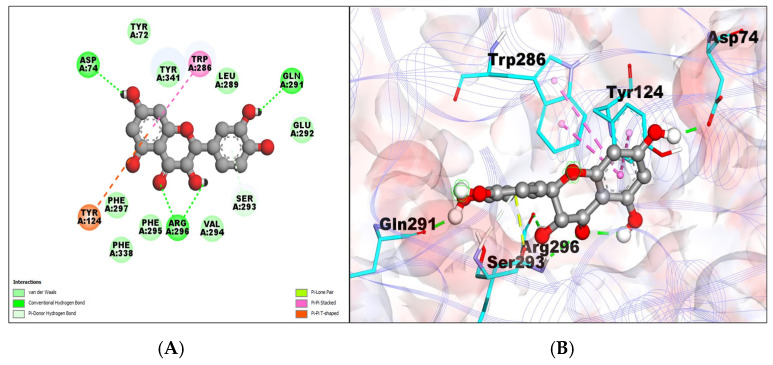
(**A**) A 2D representation of AChE–taxifolin (green colored ball and stick pattern in the center) interaction. The interacting amino acid residues are in the surroundings with different color spheres; furthermore, dotted lines show the formation of bonds. (**B**) A 3D representation of observed pose of AChE–taxifolin complex. AChE is shown by purple color in line ribbon pattern. Interacting amino acid residues are shown by a stick pattern (cyan color) in the surrounding area.

The AChE–taxifolin complex is shown at −8.85 kcal/mol with an inhibition constant of 326.70 nM, building five hydrogen bonds. Van der Waals interactions were created by amino acid residues TYR72, TYR341, PHE297, PHE338, PHE295, VAL294, GLU292, and LEU289.The pi–pi stack is shown by amino acid residues TYR124 and TRP286 (Table 1; Figure 2A,B).

**Figure 3:**
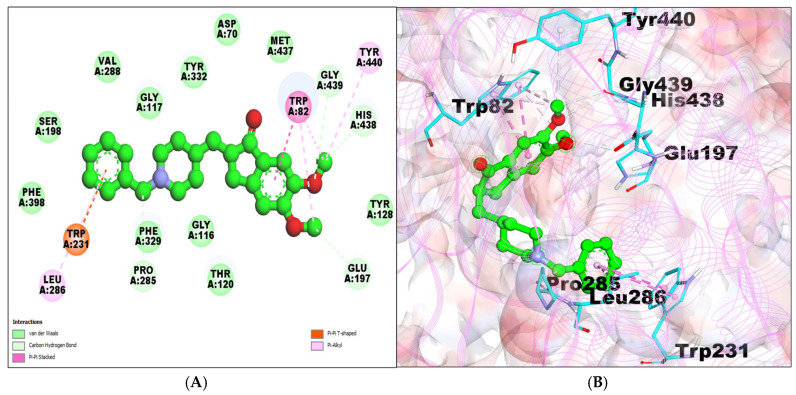
(**A**) A 2D representation of BChE–donepezil (green colored ball and stick pattern in the center) interaction. The interacting amino acid residues are in the surrounding areas with different color spheres; furthermore, dotted lines show the formation of bonds. (**B**) A 3D representation of observed pose of BChE–donepezil complex. BChE is shown by pink colored line ribbon pattern. Interacting amino acid residues are shown by a stick pattern (cyan color) in the surrounding area.

The BChE–donepezil interaction showed a −7.67 kcal/mol binding affinity with a 2.39 μM inhibition constant and formed a total of four hydrogen bonds. The amino acid residue ER198, PHE398, PHE329, GLY116, THR120, TYR128, MET437, ASP70, TYR332, GLY117, and VAL288 were involved in the van der Waals interactions. LEU286, TRP82, andTYR440 built pi–alkyl bonds, while T-Shaped/Pi–Pi stacks were formed by TRP82 and TRP231 (Table 1; Figure 3A,B).

**Figure 4:**
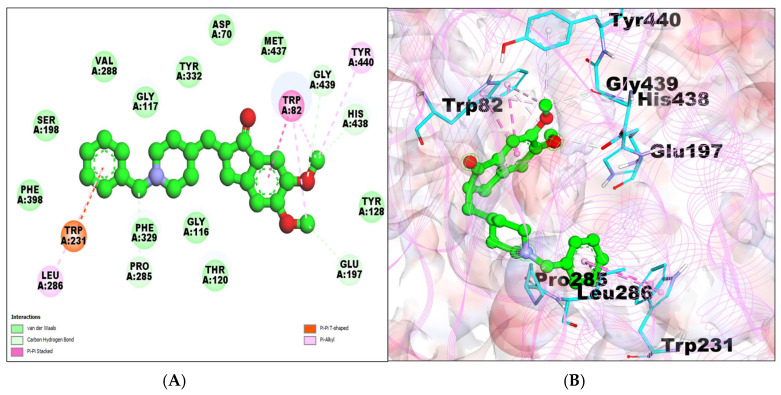
(**A**) A 2D representation of BChE–taxifolin (green colored ball and stick pattern in the center) interaction. The interacting amino acid residues are in the surrounding areas, shown by different colored spheres; furthermore, dotted lines show the formation of bonds. (**B**) A 3D representation of observed pose of BChE–taxifolin complex. BChE is shown by pink colored line ribbon pattern. Interacting amino acid residues are shown by a stick pattern (cyan color) in the surrounding area.

The BChE–taxifolin complex showed a binding energy of −7.42 kcal/mol and a 3.65 μM inhibition constant, forming seven hydrogen bonds. Taxifolin also formed van der Waals interactions with the amino acid residues of BChE, namely, ILE69, ASN68, GLY121, SER79, TRP430, MET437, GLY439, and TYR440. Pi–alkylALA328 and pi–pi stacks were formed by TRP82 (Table 1; Figure 4A,B).

Donepezil was observed to bind with AChE through two hydrogen bonds: one hydrogen bond between the carboxylate group of the Donepezil molecule and the amino group of a lysine residue and the other between the amino group of the Donepezil moiety’s group and an aspartic acid residue’s carbonyl group. Additionally, donepezil was observed to create van der Waals interactions with several AChE residues, including the side chains of the phenylalanine, tyrosine, and leucine residues. Taxifolin interacts with AChE by forming a single hydrogen bond with AChE’s hydroxyl group and the aspartic acid’s acid group. Taxifolin has also been reported to display varied hydrophobic bonding, mainly viavan der Waals forces [18], which result from interactions with several AChE residues, including tyrosine, phenylalanine side chains, and leucine residues. Not only do taxifolin and donepezil interact with AChE and BChE via van der Waals interactions and hydrogen bonding, but they have also interacted in other ways. For instance, donepezil interacts with an AChE tyrosine residue via a pi–pi stacking interaction. An alanine residue in BChE interacts with taxifolin through a pi–alkyl reaction.

It is most likely that donepezil and taxifolin have varied binding affinities for AChE and BChE because of the various structural characteristics of these compounds. AChE can generate more hydrogen bonds and van der Waals interactions with the bigger Donepezil moiety in donepezil. Due to its smaller moiety, AChE generates fewer hydrogen bonds and van der Waals interactions with taxifolin [19]. The therapeutic efficacy of donepezil and taxifolin may be affected by their differing AChE and BChE binding affinities. Alzheimer’s disease is an illness marked by the buildup of amyloid plaques in the brain. Donepezil, a drug with a greater binding capacity for AChE, may be more successful in treating this condition.

Myasthenia gravis, a disorder of the nervous system that involves the weakening and exhaustion of muscles in the skeletal system, maybe more effectively treated with taxifolin, which has a greater binding affinity for BChE [20]. The outcomes demonstrate the efficiency of donepezil and taxifolin as AChE and BChE inhibitors. Taxifolin was observed to have high negative binding energy, meaning it could be more effective than donepezil. However, donepezil is more effective at treating AChE. In general, the evidence points to donepezil and taxifolin as potential treatments for people with Alzheimer’s disease and myasthenia gravis. A further investigation is required to verify these results and establish the best administration times and doses for these substances.

### 2.2. Absorption, Distribution, Metabolism, Elimination, and Toxicity (ADMET) Analysis

ADMET are pharmacokinetics parameters which provide kinetic information about a drug’s molecules. In this study, computational models were used to predict a drug’s kinetics ADMET. This knowledge can be employed to find possible alternative medication and improve novel drug designs. A drug’s effectiveness and safety can be determined by its ADMET characteristics. For instance, a medication that is poorly absorbed into the bloodstream will not be able to reach its intended areas. If a medicine is digested too quickly, it will not have the time to produce its intended therapeutic impact [21]. Additionally, a poisonous medicine will affect the body. The success and effectiveness of drug development can be increased by using silico ADMET analysis, which is a useful tool. However, it is crucial to remember that these simulations are not ideal and should not be utilized in place of animal experimentation. The prediction of pharmacological parameters from SwissADME for taxifolin and donepezil is shown in Supplementary Table S1.

Both donepezil and taxifolin are anticipated to have good Gastrointestinal (GI) absorption. This is significant because both medications are meant to be taken orally [22]. According to predictions, P-glycoprotein (Pgp), a protein that can efflux medicines out of cells, is not projected to be a base for either taxifolin or donepezil. P-glycoprotein (Pgp) substrates, do not include taxifolin or donepezil. This is crucial for both medications because Pgp might decrease the amount of medication that reaches its intended tissues. Taxifolin and donepezil are not anticipated to inhibit CYP1A2, an enzyme that breaks down various molecules. This is essential for both medications because CYP1A2 suppression can raise the concentrations of other medications in the body, resulting in toxicity. Although donepezil is anticipated to be a CYP2C19 inhibitor, taxifolin is not. This is significant given that CYP2C19 is the main enzyme responsible for the metabolism of donepezil. The body’s donepezil levels may rise due to CYP2C19 inhibition, which could worsen the drug’s negative effects. This is significant for donepezil because the main enzyme responsible for the metabolism of many medicines is CYP2C9. In contrast to taxifolin, donepezil is not projected to be a CYP2D6 inhibitor. This is significant for donepezil because the main enzyme responsible for the metabolism of many medicines is CYP2D6.

Moreover, donepezil is likely to be a CYP3A4 inhibitor, while taxifolin is not. This is significant for donepezil because the main enzyme responsible for the metabolism of many medicines is CYP3A4. Inhibiting CYP2C9, CYP2D6, and CYP3A4 may result in higher levels of certain medications in the blood, which could be hazardous. A molecule’s skin permeability can be determined by its log Kp value. The molecule is more likely to penetrate the membrane if the log Kp value is higher. Donepezil has a positive log Kp value compared to taxifolin, which has a negative log Kp value. This indicates that donepezil is more likely to penetrate the skin, causing sensations.

In summary, SwissADME’s ADME calculations imply that donepezil and taxifolin have various physical characteristics. Compared to donepezil, taxifolin is less likely to pass through the blood–brain barrier and is more likely to be absorbed from the GI tract. Taxifolin appears less likely to inhibit CYP2C19, CYP1A2, CYP2C9, CYP3A4, and CYP2D6 than donepezil (Supplementary Table S2). Taxifolin and donepezil have a molecular weight (MW) of 304.25 and 379.49, respectively. Both substances meet the desired range of 150 to 500 g/mol. One rotatable bond exists in taxifolin, compared to six in donepezil. The required range is no more than nine rotatable bonds and seven H-bond acceptors. The preferred range is 0 to 10 H-bond acceptors. Compared to donepezil, taxifolin has five H-bond donors. The preferred range is 0–10 H-bond donors. Taxifolin has a 127.45-square-meter total polar surface area (TPSA), whereas donepezil’s 38.77 Å^2^. A range of 20 to 130 is required Å^2^. Taxifolin has a consensus log P of 0.63, while donepezil’s is 4.00. The preferred range does not exceed 6. In comparison to donepezil, taxifolin exhibits no Lipinski violations, zero Veber infractions, no Muegge violations, no Egan violations and zero Ghose infractions. Zero infractions are the target number. Taxifolin has a bioavailability score of 0.55. A score of at least 0.25 is desired. Taxifolin has a synthetic accessibility of 3.51, while donepezil has 3.36. The target value is normal between 1 (easy synthesis) and 10 (extremely difficult synthesis). According to the SwissADME’s drug-likeness predictions, taxifolin and donepezil are not very similar. Compared to donepezil, taxifolin is less rotatable, smaller, and has a greater TPSA. Additionally, compared to donepezil, taxifolin had fewer Ghose violations, Lipinski violations, Veber violations, Muegge violations, and Egan violations. It is crucial to remember that these are just forecasts. The real characteristics of a medicine may differ from the estimates. Furthermore, only a few data points are used to generate these forecasts. The precision of these forecasts will probably increase as more data becomes accessible. The toxicity predictions from pkCSM for taxifolin and donepezil are shown in Supplementary Table S3.

The test developed by *Salmonella typhimurium* reverse mutation assay (AMES) can identify possible carcinogens by using bacterial mutagenesis. Donepezil is oncogenic and mutagenic, as determined by using the AMES test, while taxifolin is not. There is a cardiac ion channel known as the hERG potassium channel that plays a significant role in the heart’s electrical activity. Torsades de pointes is a life-threatening arrhythmia that can occur when hERG channels are blocked. Contrary to donepezil, taxifolin does not block hERG I channel. Moreover, the hERG II potassium channel is an ion channel in the heart that resembles hERG I channel, and its blockage results in arrhythmias. Donepezilin inhibits hERG II channels, while taxifolin does not. Oral rat acute toxicity (LD50) is the amount of a substance that will cause the death of 50% of rats if administered orally. Donepezil’s LD50 in rats is 3.102 mg/kg, and Taxofolin’s LD50 in rats is 2.261 mg/kg. The lowest observed adverse effect level (LOAEL) for Oral Rat Chronic Toxicity is the dose at which adverse effects become noticeable in a chronic toxicity study. Taxifolin’s LOAEL in rats is 3.102 mg/kg, while donepezil’s is 0.991 mg/kg. Unlike donepezil, taxifolin is not hepatotoxic. While donepezil is a skin sensitizer, taxifolin is not. Donepezil is poisonous to *T. pyriformis*, minnows, whereas taxifolin is not. The results of pkCSM’s toxicity estimations indicate that the toxicological characteristics of donepezil and taxifolin are distinct. Thus, in summary, Donepezil is mutagenic, hepatotoxic, a skin sensitizer, has a smaller LD50 in rats, inhibits hERG pathways, is toxic to *T. pyriformis* and minnows, and has a lower MTD in humans. It is crucial to remember that these are forecasts. A drug’s real toxicity may differ from estimates. Furthermore, only a few data points are used to generate these forecasts. The precision of these forecasts will probably increase as more data becomes accessible.

### 2.3. Molecular Dynamics Simulation(MDS) Analyses

**Figure 5:**
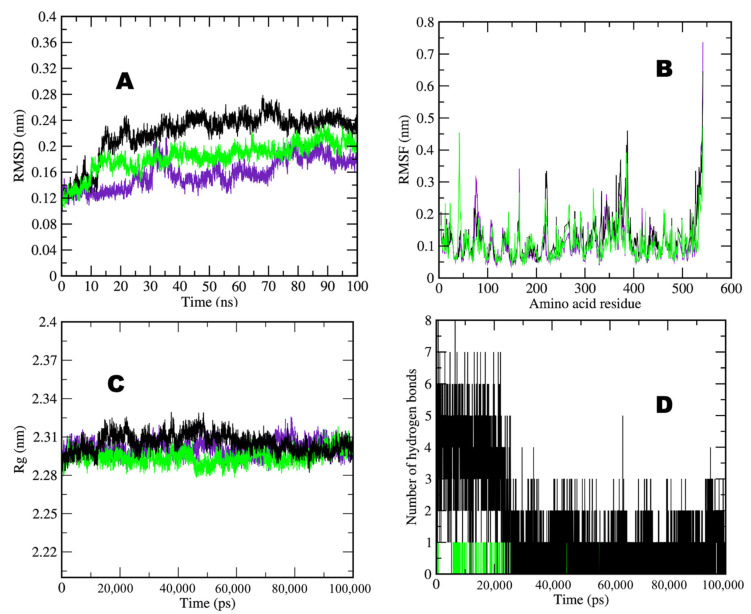
Two-dimensional graphs showing (**A**) RMSD plot of AChE in water (purple), AChE–taxifolin (black), and AChE–donepezil (green) deviation during 100 ns period; (**B**) RMSF plot with fluctuation per residues; (**C**) radius of gyration (Rg) plot showing compactness of protease molecule during 100 ns simulation; and (**D**) hydrogen bond plot showing formation hydrogen bond. Where nm = nanometer; ps = picosecond. AChE:Purple; AChE-Donzepil: green; AChE-Taxifolin: black.

After completing 100 ns of the molecular dynamics simulation, analyses were extracted from trajectory files containing Root Mean Square Deviation (RMSD), Root Mean Square Fluctuation (RMSF), Radius of Gyration (Rg), and the formation of numbers of hydrogen bond data—the deviation of both complexes and AChE and BChE in water during the 100 ns MDS. The average RMSD values observed for AChE in water, AChE–taxifolin, and AChE–donepezil were between 0.12 and 0.24 nm for complexes and AChE simulations in water (Figure 5A). Significantly, it was observed that the AChE–taxifolin complex showed an average value near 0.2 nm, and the AChE–donepezil complex showed approximately average values near 0.16 nm with stability, which is higher than the AChE simulation in water, i.e., approximately 0.12 nm. An RMSF calculation per residue shows all the overall values for the complexes, which were between 0.1–0.7 nm (Figure 5B). The observed average value was less than 0.1 nm for all simulated molecules. Selected complexes, including the AChE simulation in water, showed similar fluctuation patterns except for some significant fluctuations observed at the 50–80, 150–160, 210–230, and 350–380 amino acid regions. The radius of the gyration analysis is very important for assessing the compactness and stability of protein structures during the simulation period because of the presence of ligand molecules. The observed average value of Rg ranged between 2.28 and 2.31 nm for all complexes. AChE in water and AChE–taxifolin showed an average value of 2.31 nm, while the control AChE–donepezil showed an approximate value ranging near to 2 (Figure 5C). One to eight hydrogen bonds formed during selected compounds and the AChE receptor interaction (Figure 5D). AChE–taxifolin formed eight hydrogen bonds, while control AChE–donepezil formed only one hydrogen bond during the simulation period.

**Figure 6:**
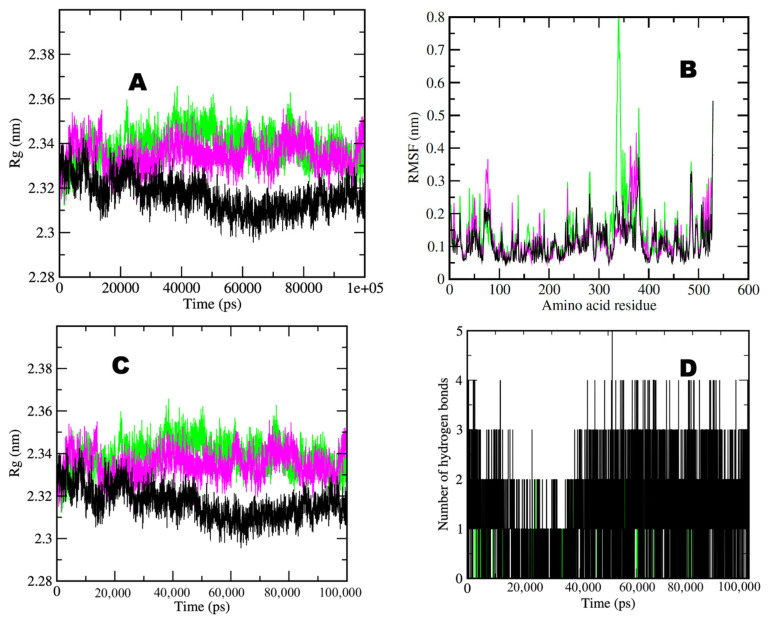
Two-dimensional graphs showing (**A**) RMSD plot of BChE in water (pink), BChE–taxifolin (black), and BChE–donepezil (green) deviation during 100 ns period; (**B**) RMSF plot with fluctuation per residues; (**C**) radius of gyration (Rg) plot showing compactness of protease molecule during 100 ns simulation; and (**D**) hydrogen bond plot showing formation hydrogen bond. Where nm = nanometer; ps = picosecond. AChE:Purple; AChE-Donzepil: green; AChE-Taxifolin: black.

Also, BChE simulation results were analyzed to check the stability of the complex. The average RMSD values observed for BChE in water, BChE–taxifolin, and BChE–donepezil was between 0.15 and 0.3 nm for complexes (Figure 6A). Significantly, it was observed that the BChE–taxifolin complex showed an average value of 0.5 nm, which is less than the average value of 0.3 nm observed for the control BChE–donepezil complex. In comparison, simulation in water showed a value of approximately 0.2 nm for the whole simulation period. RMSF calculation per residue shows the overall values for all complexes, which were between 0.1 and 0.8 nm (Figure 6B). The observed average value was 0.1 nm for all simulated molecules. Selected complexes, including the BChE simulation in water, showed similar fluctuation patterns except for some major fluctuations observed at the 70–85, 150–160, 280–300, and 320–370 amino acid regions. The observed average value of Rg ranged between 2.32 and 2.34 nm for all complexes. BChE in water and control BChE–donepezil showed an average value near 2.34 nm, while BChE–taxifolin showed an approximate value less than the control, i.e., 2.32 nm (Figure 6C). Between one and four hydrogen bonds formed during selected compounds and BChE receptor interactions (Figure 6D). BChE–taxifolin formed one to four hydrogen bonds, while the control BChE–donepezil formed only one to two hydrogen bonds during the simulation period.

MDS trajectory files were further subjected to Molecular Mechanics Poisson-Boltzmann Surface Area (MMPBSA) analysis. The data obtained from Poisson–Boltzmann complex energy and ligand–receptor energy component calculations are presented in Table 2 and Table 3. The table shows the results of a free energy calculation for binding taxifolin to two different enzymes, AChE and BChE. Free energy is calculated as the sum of several components, including the van der Waals energy, electrostatic energy, polar solvation energy, dispersion energy, gas phase energy, and solvent energy. The results show that the binding of taxifolin to AChE is more favorable than to BChE. The total free energy of binding for AChE–taxifolin is −33,414.08 kcal/mol, while the total free energy for BChE–taxifolin is −35,219.97 kcal/mol. This difference is due to several factors, including the stronger van der Waals interactions between taxifolin and AChE, the more favorable electrostatic interactions between taxifolin and AChE, and the more favorable polar solvation of taxifolin by water. The stronger van der Waals interactions between taxifolin and AChE are likely due to the larger surface area of taxifolin. The more favorable electrostatic interactions between taxifolin and AChE are likely due to the positive charge on the AChE binding pocket. The more favorable polar solvation of taxifolin by water is likely due to the former’s polar nature. These results suggest that taxifolin is a more effective inhibitor of AChE than BChE, which is consistent with its known pharmacological properties. Furthermore, taxifolin is a natural product shown to have anti-inflammatory and anti-cancer properties. The results of this free energy calculation provide a quantitative explanation for the difference in binding affinity between taxifolin and BChE. The stronger van der Waals interactions, the more favorable electrostatic interactions, and the more favorable polar solvation of taxifolin all contribute to its greater binding affinity. These results could be used to design new drugs that are more effective inhibitors of AChE and BChE (Table 2).The total free energy of binding for AChE–taxifolin is −24.34 kcal/mol, while the total free energy of binding for BChE–taxifolin is −16.14 kcal/mol.

## 3. Methodology

### 3.1. Ligand Preparation

We obtained the 2D structures and SMILES IDs of taxifolin (https://pubchem.ncbi.nlm.nih.gov/compound/439533) (accessed on 22 May 2023) and the control drug donepezil (https://pubchem.ncbi.nlm.nih.gov/compound/3152) (accessed on 22 May 2023) from the PubChem database [23,24].

We then used the Novoprolab server (https://www.novoprolabs.com/tools/smiles2pdb) (accessed on 22 May 2023) to convert the SMILES IDs into 3D Protein Data Bank (PDB) files [25,26] for subsequent molecular docking and simulation studies. Next, we submitted the ligand files to the Discovery Studio visualize version 21.1.0.20298 to perform energy minimization [26,27,28]. Using empirical energy functions, we applied the CHARMm forcefield to model the macromolecular systems [29].

### 3.2. Receptor Preparation

AChE and BChE are enzymes that hydrolyze choline esters. AChE is the most abundant cholinesterase in the body and is responsible for the hydrolysis of the neurotransmitter acetylcholine. BChE is less abundant than AChE and is responsible for the hydrolysis of butyrylcholine and other choline esters. AChE and BChE are important enzymes that play a role in nerve signaling. AChE is found in the nervous system, red blood cells, and the placenta [30]. BChE is found in the liver, intestine, and plasma. Inhibitors of AChE and BChE are used to treat various conditions, including Alzheimer’s disease, myasthenia gravis, and glaucoma [31].Therefore, we have chosen both enzymes as receptors for investigation. PDB ID: 7E3H is the PDB identifier for the crystal structure of human AChE in complex with donepezil, a drug used to treat Alzheimer’s disease. X-ray crystallography determined the structure with a resolution of 2.45 Å [32].

PDB ID: 7AIY is the crystal structure of human BChE in complex with 2-{1-[4-(12-Amino-3-chloro-6,7,10,11-tetrahydro-7,11-methanocycloocta[b]quinolin-9-yl) butyl]-1H-1,2,3-triazol-4-yl}-N-[4-hydroxy-3-methoxybenzyl] acetamide. The structure was determined with X-ray crystallography and has a resolution of 2.94 Å [33]. The next step involves removing any water molecules, cofactors, or other unwanted molecules from the receptor structure, adding hydrogen atoms, assigning charges and atom types, and optimizing the geometry produced by Discovery Studio Visualizer version 21.1.0.20298. This step involves finding the receptor region, where the ligand can bind and interact with its amino acid residues. We analyzed the binding site of both PDB structures by using Discovery Studio Visualizer version 21.1.0.20298 described by Biovia, 2021 [26]. The key amino acids were identified and considered for the active site docking of selected natural compounds.

### 3.3. AutoDock 4.2 Tool Receptor-Ligand Docking

In AutoDock version 4.2, water molecules, cofactors, or other unwanted molecules from the receptor structure were removed, and we added hydrogen atoms, assigned charges and atom types, and optimized the geometry. Different conformations and tautomers must be generated for the ligand so that it can fit into the receptor binding site. Furthermore, we set up the grid box that covers the region of interest where the docking is performed. For AChE, the grid point in x, y, and z was 60 × 60 × 60, with spacing 0.375, and the grid center x, y, and z co-ordinates were −40.919, 35.841, and −29.382, respectively, while the grid center x, y, and z co-ordinates for BChE were −21.246, −11.966, and 44.337. The grid box defines the size and resolution of the grid points used to calculate the interaction energies between the receptor and the ligand [34,35]. The Lamarckian Genetic Algorithm (LGA) determines how the ligand is placed and rotated in the receptor binding site. The scoring function evaluates how well the ligand fits into the receptor and estimates its binding affinity(ΔG), as per the following formula:

ΔG_binding_ = ΔG_gauss_ + ΔG_repulsion_ + ΔG_hbond_ + ΔG_hydrophobic_ + ΔG_tors_,

where ΔG_gauss_ is an attractive term for the dispersion of two Gaussian functions; ΔG_repulsion_ is the square of the distance if it is closer than a threshold value; ΔG_hbond_ is a ramp function that is also used for interactions with metal ions; ΔG_hydrophobic_ is a ramp function; and ΔG_tors_ is proportional to the number of rotatable bonds. The parameters for population size (ga_pop_size) 150; energy evaluations (ga_num_evals) 2,500,000; maximum number of generations (ga_num_generations) 27,000; mutation rate 0.02; crossover rate 0.8; beta parameter Cauchy distribution (ga_cauchy_beta) 1.0; probability of performing local search on individual (ls_search_freq) 0.6; and the LGA executions were maximized to 10 runs.

Finally, the AutoDock version 4.2 program executed the provided parameter after the successful run, and prepared receptor and ligand files, as well as the defined docking grid and parameters, depending on the size and complexity of the receptor and the ligand. In the last examination of the docking poses, we ranked them according to their scores, visualized them using molecular graphics software, and compared them with control data using Discover Studio Visualizer version 21.1.0.20298 [26].

### 3.4. Drug-Likeness and ADMET

We used the SwissADME online tool (http://www.swissadme.ch) (accessed on 12 October 2023) from the Swiss Institute of Bioinformatics (SIB), Lausanne, Switzerland [36] to computationally predict the ADME, drug-likeness, and pharmacokinetics properties of the selected natural compounds. This tool calculated parameters for physicochemical properties like molecular weight, hydrogen bond donors/acceptors, number of rotatable bonds, and topological polar surface area (TPSA). For lipophilicity, it can calculate parameters like Consensus logP. Pharmacokinetic parameters calculations include gastrointestinal absorption, blood–brain barrier (BBB) permeability, P-glycoprotein (P-gp) substrate, cytochrome P450 (CYP) inhibition, and bioavailability. Drug-likeness parameters include Lipinski’s rule of five (Ro5), Veber’s rule, and bioavailability score. We also analyzed additional toxicity using the pkCSM online server (http://biosig.unimelb.edu.au/pkcsm/) (accessed on 15 October 2023). pkCSM calculates several toxicity parameters, such as AMES mutagenicity, and predicts whether a molecule is likely to cause mutations in bacteria. Carcinogenicity predicts the potential for a molecule to cause cancer in rodents. The human ether-a-go-go-related gene (hERG) inhibition parameter predicts whether a molecule is likely to block the hERG potassium channel, which can lead to cardiac arrhythmias. Oral rat acute toxicity (LD50) calculation estimates the dose of a drug that is lethal to 50% of rats.

### 3.5. Molecular Dynamics Simulations (MDS)

We performed 100 ns MDS for the selected complexes of AChE–donepezil, AChE–taxifolin, BChE–donepezil, and BChE–taxifolin using the GROMACS tool 2018 version [37] from the University of Groningen, The Netherlands. We also simulated AChE and BChE in water for comparison. We used the pdb2gmx module to generate the topology files of each corresponding protein in complex while employing the CHARMM27 all-atom force field. In parallel, the topology file of each corresponding ligand was generated using the SwissParam server (http://www.swissparam.ch/) (accessed on 18 October 2023). We created a triclinic box unit cell filled with TIP3P water model for solvation and added Na^+^ and Cl^−^ ions to stabilize the system. We minimized energy by employing a maximum force constraint of 10 kJ/mol to prevent potential steric hindrance. The equilibration of each system was obtained by using two-step ensembles. We applied the NVT (constant number of particles, pressure, and temperature) ensemble at 300 K for 100 ps using V-rescale thermostat to achieve temperature equilibration. The NPT (constant number of particles, pressure, and temperature) of each of system was equilibrated by using a Parrinello-Rahman barostat [38,39]. During simulation, particle mesh ewald (PME) was used to estimate long-range electrostatic interactions. Bond lengths were restrained by LINear Constraint Solver (LINCS) algorithm [40].

## 4. Conclusions

Numerous plants have been found to exhibit cholinesterase inhibitory activity, indicating they can protect nerve cells by delaying the depletion of the neurotransmitter acetylcholine, which is crucial for memory and cognition. Taxifolin’s significant binding energy with both tested enzymes indicated its potentiality to inhibit AChE and BChE. The dynamics and simulation study results indicated that taxifolin could be safer and more strongly bind and inhibit AChE and BChE than standard drugs. The inhibition of AChE and BChE by taxifolin is likely due to its ability to bind to their active sites, which could have therapeutic implications for the treatment of Alzheimer’s disease and other neurodegenerative disorders. However, more in vitro and in vivo investigations are required to verify these results to determine the safety and effectiveness of taxifolin so that it may one day be used to treat neuron-associated diseases and disorders.

## Figures and Tables

**Table 1:** Docking results obtained from AutoDock version 4.2 tool after performing molecular interaction between AChE, BChE, and the control drugs donepezil and taxifolin. In hydrogen bond column where UNK1 and UNL1 are selected ligand compounds.

Complex	Binding Energy (Kcal/mol)	Inhibition Constant (Ki) μM: micro molar	Hydrogen Bonds	Hydrogen Bond Length Å(Angstrom)	Van der Waals Interaction	Other Interaction
AChE–donepezil (Control)	−9.33	0.144 μM	UNK1:DNP:C28–A:ASP74:OD1	3.03318	TYR72,TYR341, LEU289,GLU292, VAL294,PHE295, PHE338,PHE297	PI–PI STACKED/ PI–PI T-SHAPED TYR124, TRP286
AChE–taxifolin (PDB:7E3H)	−8.85	0.327 μM	A:ARG296:HN–:UNL1:O1	2.25805	TYR72,TYR341, PHE297,PHE338, PHE295,VAL294, GLU292,LEU289,	PI–PI T-SHAPED/PI–PI STACKED TYR124, TRP286
			UNL1:H11–A:GLN291:O	1.87707		
			UNL1:H10–A:ARG296:O	2.16795		
			UNL1:H8–A:ASP74:OD2	1.96554		
			A:SER293:HN–:UNL1	2.9256		
BChE–donepezil (Control)	−7.67	2.390 μM	A:GLY439:CA–UNK1:E20601:O25	3.55463	SER198,PHE398, PHE329,GLY116, THR120,TYR128, MET437,ASP70, TYR332,GLY117, VAL288	PI–ALKYL LEU286,TRP82, TYR440 PI–PI T-SHAPED/PI–PI STACKED TRP82,TRP231
			UNK1:E20601:C17–A:PRO285:O	3.19904		
			UNK1:E20601:C26–A:HIS438:O	2.85071		
			UNK1:E20601:C28–A:GLU197:OE1	3.18095		
BChE–taxifolin (PDB:7AIY)	−7.42	3.650 μM	A:TYR332:HH–:UNL1:O1	2.12817	ILE69,ASN68, GLY121,SER79, TRP430,MET437, GLY439,TYR440	PI–ALKYL ALA328 PI–PI STACKED TRP82
			UNL1:H12–A:GLN67:OE1	1.99252		
			UNL1:H11–A:ASN83:OD1	1.91515		
			UNL1:H10–A:ASP70:OD1	1.83371		
			UNL1:H8–A:HIS438:O	2.22385		
			A:PRO84:CD–:UNL1:O7	3.47997		
			A:HIS438:CD2–:UNL1:O3	2.97375		

**Table 2:** Representing the summarized data of Poisson–Boltzmann complex energy components calculation with ± SEM (standard deviation error of the mean) of complexes AChE–donepezil, AChE–taxifolin, BChE–donepezil, and BChE–taxifolin. Where ΔVdwaals = van der Waals energy; ΔEEL = electrostatic molecular energy; ΔEPB = polar contribution to the solvation energy; ΔENPOLAR = nonpolar contribution of repulsive solute–solvent interactions to the solvation energy; ΔEDISPER = nonpolar contribution of attractive solute–solvent interactions to the solvation energy; ΔGGas = total gas phase molecular energy; ΔGSolv = total solvation energy; and ΔGTotal = total binding energy.

Complex Free Energy Calculation Components (kcal/mol)								
Complex	ΔVdwaals	ΔEEL	ΔEPB	ΔENPOLAR	ΔEDISPER	∆GGas	∆GSolv	∆GTotal
AChE–donepezil	−4325.87 (±5.40)	−29,859.62 (±21.16)	−4848.75 (±19.85)	102.59 (±0.23)	0.00 (±0.0)	−28,931.46 (±22.49)	−4746.16 (±19.71)	−33,677.61 (±11.03)
AChE–taxifolin	−4313.25 (±5.17)	−29,618.25 (±16.14)	−5040.32 (±15.79)	104.17 (±0.24)	0.00 (±0.0)	−28,477.93 (±18.85)	−4936.15 (±15.64)	−33,414.08 (±9.20)
BChE–donepezil	−4372.48 (±5.80)	−32,642.66 (±23.70)	−6031.58 (±22.45)	112.68 (±0.24)	0.00 (±0.00)	−29,327.66 (±25.01)	−5918.90 (±22.30)	−35,246.56 (±14.62)
BChE–taxifolin	−4398.54 (±4.41)	−32,414.36 (±20.44)	−6199.93 (±20.18)	110.48 (±0.23)	0.00 (±0.00)	−29,130.52 (±18.68)	−6089.45 (±20.06)	−35,219.97 (±13.65)

**Table 3:** Representing the summarized data of MMPBSA-based free energy calculation components with ± SEM (standard deviation error of the mean) of ligand–receptor. Where ΔVdwaals = van der Waals energy; ΔEEL = electrostatic molecular energy; ΔEPB = polar contribution to the solvation energy; ENPOLAR = nonpolar contribution of repulsive solute–solvent interactions to the solvation energy; ΔEDISPER = nonpolar contribution of attractive solute–solvent interactions to the solvation energy; ΔGGas = total gas phase molecular energy; ΔGSolv = total solvation energy; and ΔGTotal = total binding energy.

Ligand–Receptor Free Energy Calculation Components								
Complex	ΔVdwaals	ΔEEL	ΔEPB	ΔENPOLAR	ΔEDISPER	∆GGas	∆GSolv	∆GTotal
AChE–donepezil	−56.23 (±0.46)	−256.40 (±1.50)	282.05 (±2.05)	−5.09 (±0.02)	0.00 (±0.00)	−312.62 (±1.70)	276.96 (±2.04)	−35.66 (±0.93)
AChE–taxifolin	−35.49 (±0.37)	−10.23 (±0.71)	24.44 (±0.73)	−3.06 (±0.02)	0.00 (±0.00)	−45.72 (±0.80)	21.38 (±0.80)	−24.34 (±0.56)
BChE–donepezil	−44.72 (±0.47)	−166.47 (±2.63)	182.10 (±2.34)	−4.80 (±0.02)	0.00 (±0.00)	−211.19 (±2.77)	177.30 (±2.33)	−33.90 (±0.73)
BChE–taxifolin	−34.75 (±0.37)	−8.99 (±0.53)	31.02 (±0.59)	−3.42 (±0.02)	0.00 (±0.00)	−43.74 (±0.64)	27.60 (±0.58)	−16.14 (±0.52)

## Data Availability

Data are contained within the article and Supplementary Materials.

## Supplementary Materials

The following supporting information can be downloaded at: https://www.mdpi.com/article/10.3390/molecules29030674/s1, Table S1: ADME prediction from SwissADME (GI = Gastro intestinal, BBB = Blood Brain Barrier, Pgp = P glycoprotein, CYP = Cytochrome, log Kp = skin permeation); Table S2: Drug-likeness prediction from SwissADME server (MW = Molecular Weight, TPSA = total polar surface area, Consensus Log P = average of all predicted Log Po/w; Table S3: toxicity prediction. Data obtained from pkCSM server.
