# Supplementary material for: Computational Approaches to Evaluate the Acetylcholinesterase Binding Interaction with Taxifolin for the Management of Alzheimer’s Disease
Source: Molecules. 2024 Jan 31;29(3):674. doi: 10.3390/molecules29030674 (PMC10856623; doi:10.3390/molecules29030674)
Supplement: Supplementary file 1 [file molecules-29-00674-s001.zip › molecules-2763343-supplementary.pdf]

Table S1. ADME prediction from SwissADME (GI=Gastro intestinal, BBB=Blood Brain Barrier, Pgp=P glycoprotein, CYP=Cytochrome, log Kp= skin permeation)

**Compounds**	**GI absorption**	**BBB permeant**	**Pgp substrate**	**CYP1A2 inhibitor**	**CYP2C19 inhibitor**	**CYP2C9 inhibitor**	**CYP2D6 inhibitor**	**CYP3A4 inhibitor**	**log Kp (cm/s)**
									negative the log K_p the less skin permeant the molecule
Taxifolin	High	No	No	No	No	No	No	No	-7.48
Donepezil	High	Yes	Yes	No	No	No	Yes	Yes	-5.58

Table S2. Drug-likeness prediction from SwissADME server (MW=Molecular Weight, TPSA= total polar surface area, Consensus Log P= average of all predicted Log Po/w													
**Compounds**	**MW (g/mol)**	**Rotatable bonds**	**H-bond acceptors**	**H-bond donors**	**TPSA (Å²)**	**Consensus Log P**	**Lipinski violations**	**Ghose violations**	**Veber violations**	**Egan violations**	**Muegge violations**	**Bioavailability Score**	**Synthetic Accessibility**
	MW between 150 and 500 g/mol	no more than 9 rotatable bonds			TPSA between 20 and 130 Å²	not higher than 6						not less than 0.25	normalized between 1 (easy synthesis) and 10 (very difficult synthesis)
Taxifolin	304.25	1	7	5	127.45	0.63	0	0	0	0	0	0.55	3.51
Donepezil	379.49	6	4	0	38.77	4.00	0	0	0	0	0	0.55	3.36

Table S3: toxicity prediction. Data obtained from pkCSM server

	AMES toxicity	Max. tolerated dose (Human)	hERG I inhibitor	hERG II inhibitor	Oral Rat Acute Toxicity (LD50)	Oral Rat Chronic Toxicity (LOAEL)	Hepatotoxicity	Skin sensitisation	T. pyriformis toxicity	Minnow toxicity
**Standard cut-off**	**Positive mutagenic**	**<= 0.477 log(mg/kg/day)**				**lowest**			**> -0.5 log ug/L**	**Log LC50< -0.3**
**Compounds**										
**Taxifolin**	No	0.345	No	No	2.261	3.102	No	No	0.286	4.688
**Donepezil**	No	-0.217	No	Yes	2.753	0.991	Yes	No	0.804	-2.011
